# Supplementary figures and images for: Phylogenetic evidence for the invasion of a commercialized European Phasmarhabditis hermaphrodita lineage into North America and New Zealand
Source: PLoS One. 2020 Aug 17;15(8):e0237249. doi: 10.1371/journal.pone.0237249 (PMC7430733; doi:10.1371/journal.pone.0237249)

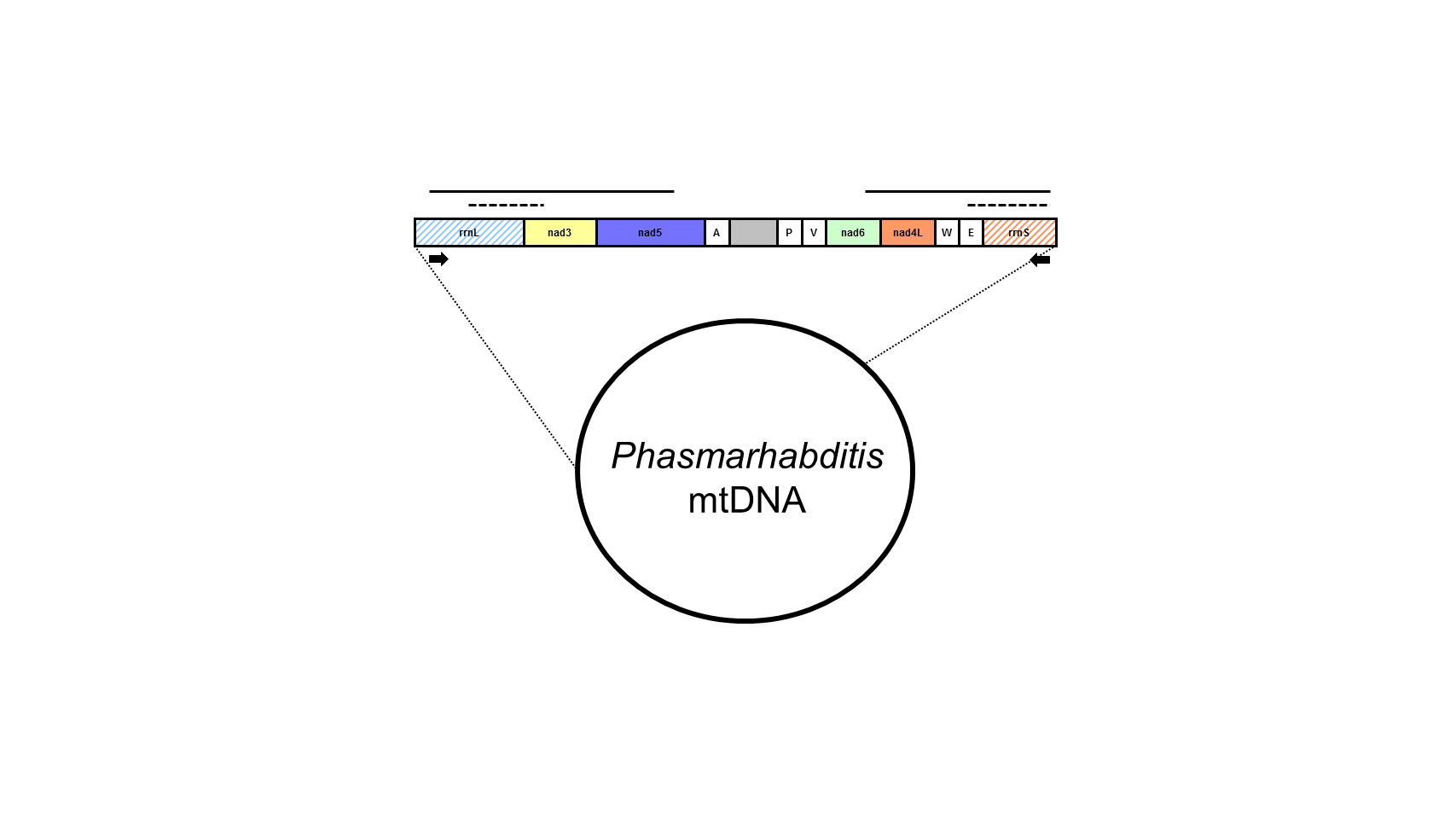

Supplement: S1 Fig — Linear representation of the fragment of mtDNA amplified using Expand Long Range PCR kit (Roche) with protein-coding genes, ribosomal RNA genes and tRNA genes noted. PCR primers, shown as large arrows, and internal species-specific primers were used for sequencing (see S1 Table). Dashed black lines indicate sequences included in Fig 1 analysis; solid black lines indicate sequences included in Fig 2 analysis. (TIF) [file pone.0237249.s001.tif]
