# Supplementary material for: Phylogenetic evidence for the invasion of a commercialized European Phasmarhabditis hermaphrodita lineage into North America and New Zealand
Source: PLoS One. 2020 Aug 17;15(8):e0237249. doi: 10.1371/journal.pone.0237249 (PMC7430733; doi:10.1371/journal.pone.0237249)
Supplement: S1 Table — (DOCX) [file pone.0237249.s002.docx]

**S1 Table. PCR and sequencing primers used to target *Phasmarhabditis hermaphrodita* mtDNA.**

| Primer name* | Primer sequence (5' → 3') |
| --- | --- |
| 18A | AAAGATTAAGCCATGCATG |
| 26R | CATTCTTGGCAAATGCTTTCG |
| C_lsuA_F1 | AATGGCAGTCTTAGCGTGA |
| Ph_mt01F | GGCATTTAGTACGAAAGGAAAA |
| Ph_mt03bF | TGTCTTTGTTAGAGTGGGACTTC |
| Ph_mt06R | ACCATCTTGCTGACCAAAAG |
| Ph_mt05F | ACCAAAAGCTATAAGAGCACCA |
| C_ssuB_R1 | GCAATTGATGGATGATTTG |
| Ph_mt02R | CAGAAACTAATTTTTGATTTTGAA |
| Ph_mt04R | CCGATATAAGTGGTATCCTAAACA |
| Ph_mt09F | TCTTTTTCACAGAAAAGGTAATTT |
| Ph_mt08R | TCCCACACTTACAAATCCAC |

*18S primers from Blaxter ML, De Ley P, Garey JR, Liu LX, Scheldeman P, Vierstraete A, et al. A molecular evolutionary framework for the phylum Nematoda. Nature. 1998;392(6671): 71-5.
