# Supplementary material for: Phylogenetic evidence for the invasion of a commercialized European Phasmarhabditis hermaphrodita lineage into North America and New Zealand
Source: PLoS One. 2020 Aug 17;15(8):e0237249. doi: 10.1371/journal.pone.0237249 (PMC7430733; doi:10.1371/journal.pone.0237249)
Supplement: S2 Table — (DOCX) [file pone.0237249.s003.docx]

**S2 Table. Bacteria associated with Oregon *P. hermaphrodita***

| Bacterial colony ID | Top BLAST hit | Accession  Number | % identity | E-value |
| --- | --- | --- | --- | --- |
| DL300.1 | Sphingobacterium sp. 1ZP4 16S ribosomal RNA gene, partial sequence | HQ540555.1 | 99 | 0 |
| DL300.2 | Sphingobacterium sp. 1ZP4 16S ribosomal RNA gene, partial sequence | HQ540555.1 | 99 | 0 |
| DL300.3 | Sphingobacterium sp. 1ZP4 16S ribosomal RNA gene, partial sequence | HQ540555.1 | 98 | 0 |
| DL300.4 | Pseudomonas sp. strain ACO-106 16S ribosomal RNA gene, partial sequence | KU869718.1 | 95 | 0 |
| DL300.5 | Pseudomonas sp. strain CNL-2 16S ribosomal RNA gene, partial sequence | KX384590.1 | 99 | 0 |
| DL300.6 | Pseudomonas sp. strain CNL-2 16S ribosomal RNA gene, partial sequence | KX384590.1 | 99 | 0 |
| DL300.7 | Pseudomonas putida strain Md1-34 16S ribosomal RNA gene, partial sequence | MF581440.1 | 99 | 0 |
| DL300.8 | Pseudomonas sp. strain CNL-2 16S ribosomal RNA gene, partial sequence | KX384590.2 | 99 | 0 |
| DL300.9 | Pseudomonas putida strain WXZ-6 16S ribosomal RNA gene, partial sequence | EF440609.1 | 97 | 0 |
| DL300.10 | Pseudomonas sp. strain FA3 16S ribosomal RNA gene, partial sequence | MK120443.1 | 91 | 0 |
| DL300.11 | Sphingobacterium sp. 1ZP4 16S ribosomal RNA gene, partial sequence | HQ540555.1 | 98 | 0 |
| DL300.12 | Klebsiella pneumoniae strain DWS24 16S ribosomal RNA gene, partial sequence | MH356581.1 | 98 | 0 |
| DL307.1 | Ochrobactrum anthropi strain FC6858 16S ribosomal RNA gene, partial sequence | MK208693.1 | 99 | 0 |
| DL307.2 | Ochrobactrum sp. p37(2011) 16S ribosomal RNA gene, partial sequence | HQ652576.1 | 98 | 0 |
| DL307.3 | Microbacterium foliorum strain LMP2 16S ribosomal RNA gene, partial sequence | MH479982.1 | 99 | 0 |
| DL307.4 | Ochrobactrum sp. 5-tr1 gene for 16S ribosomal RNA, partial sequence | LC384480.1 | 100 | 0 |
| DL307.5 | Ochrobactrum anthropi strain FC6858 16S ribosomal RNA gene, partial sequence | MK208693.1 | 99 | 0 |
| DL307.6 | Brucella melitensis strain BmWS93 chromosome 2, complete sequence | CP034104.1 | 99 | 0 |
| DL307.7 | Microbacterium maritypicum strain ICMP 20901 16S ribosomal RNA gene, partial sequence | MG786404.1 | 99 | 0 |
| DL307.8 | Pseudomonas putida strain 7B1 16S ribosomal RNA gene, partial sequence | MH379791.1 | 100 | 0 |
| DL307.9 | Pseudomonas putida strain L39 16S ribosomal RNA gene, partial sequence | KU179374.1 | 100 | 0 |
| DL307.10 | Acinetobacter guillouiae strain RCAM04485 16S ribosomal RNA gene, partial sequence | MF754134.1 | 100 | 0 |
| DL307.11 | Pseudomonas putida strain 7B1 16S ribosomal RNA gene, partial sequence | MH379791.1 | 99 | 0 |
| DL307.12 | Pseudomonas putida strain 7B1 16S ribosomal RNA gene, partial sequence | MH379791.1 | 100 | 0 |
| DL309.1 | Pseudomonas azotoformans strain S23_SO1T 16S ribosomal RNA gene, partial sequence | MK883104.1 | 99 | 6.00E-140 |
| DL309.2 | Pseudomonas putida partial 16S rRNA gene, isolate P3_55 | LT838160.1 | 100 | 0 |
| DL309.3 | Pseudomonas azotoformans strain D116_SP6R 16S ribosomal RNA gene, partial sequence | MK883209.1 | 100 | 4.00E-168 |
| DL309.4 | Acinetobacter sp. strain 05_2011_151_l4_sed_dir_B01 16S ribosomal RNA gene, partial sequence | MG896828.1 | 100 | 0 |
| DL309.5 | Pseudomonas putida partial 16S rRNA gene, isolate P3_55 | LT838160.1 | 100 | 0 |
| DL309.6 | Acinetobacter sp. strain Exi5-53 16S ribosomal RNA gene, partial sequence | MK235211.1 | 100 | 0 |
| DL309.7 | Stenotrophomonas sp. strain HS007R 16S ribosomal RNA gene, partial sequence | MH669536.1 | 100 | 0 |
| DL309.8 | Stenotrophomonas maltophilia clone B2.18.23 16S ribosomal RNA gene, partial sequence | AY837730.1 | 99 | 0 |
| DL309.9 | Acinetobacter sp. strain Exi5-53 16S ribosomal RNA gene, partial sequence | MK235211.1 | 100 | 0 |
| DL309.10 | Acinetobacter sp. strain AzaSpMN-M9 16S ribosomal RNA gene, partial sequence | MN258957.1 | 99 | 0 |
| DL309.11 | Acinetobacter sp. strain 05_2011_151_l4_sed_dir_B01 16S ribosomal RNA gene, partial sequence | MG896828.1 | 100 | 0 |
| DL309.12 | Acinetobacter sp. strain Exi5-53 16S ribosomal RNA gene, partial sequence | MK235211.1 | 100 | 0 |
